# Using Self-Study and Peer-to-Peer Support to Change “Sick” Care to “Health” Care: The Patient Perspective

**DOI:** 10.3389/fdgth.2020.00002

**Published:** 2020-06-04

**Authors:** Camille Nebeker, Bethany Weisberg, Eric Hekler, Michael Kurisu

**Affiliations:** ^1^Department of Family Medicine and Public Health, School of Medicine, University of California, San Diego, La Jolla, CA, United States; ^2^Center for Wireless and Population Health Systems, UC San Diego, La Jolla, CA, United States; ^3^The Design Lab, UC San Diego, La Jolla, CA, United States

**Keywords:** citizen science, N-of-1, digital health, self-tracking, participant-led research, peer-to-peer support, Research Ethics

## Abstract

**Background:** Access to digital health technologies is contributing to a paradigm shift where *sick*care may become authentic *health*care. Individuals can now access personal health data through wearable sensors, affordable lab screenings, genetic and genomic sequencing, and real-time health tracking apps. Personal health data access creates opportunities to study health indicators 24/7 and in real time. This is especially useful for patients with hard-to-diagnose or treat diseases, which led to a self-formed patient group called Project Apollo. Project Apollo is composed of highly motivated patients with common experiences of undiagnosed conditions, a lack of clear treatment options, and shared frustrations with navigating the U.S. healthcare system. These experiences have led the Apollo cohort to supplement their health knowledge through self-study research.

**Objective:** To qualify the experience and expectations of patients affiliated with Project Apollo.

**Methods:** A qualitative approach involved record review and semi-structured interviews. One-hour semi-structured interviews were conducted to solicit motivations, expectations, and potential barriers and facilitators to self-study followed by a brief survey on digital tool use. Interviews were digitally recorded, transcribed, and analyzed to identify themes and patterns.

**Results:** Participants included six females and three males ranging in age from 30 to 70+ years. Responses were organized under five key themes including: frustration with healthcare system; community support; self-study/N-of-1 research; access to experts; moving from sick to healthcare. Facilitators include motivation, albeit stemming from frustration, a safe community where patients derive support, and access to experts for guidance. Increasing awareness of clinicians about the potential value of partnering with patients who are advancing health knowledge through self-study is critical.

**Conclusions:** N-of-1 self-study research, coupled with community support and digital health tools, appears to be one plausible pathway to shifting the paradigm from *sick*care toward patient-partnered *health*care.

## Introduction

The structure and operations of healthcare in the United States (US) is grounded in prioritizing acute care over individual health promotion and disease prevention as well as public health (1). This *sick*care system was classically created to enable people to receive expert, evidence-based advice and support to help diagnose and treat diseases (1). A dominant paradigm in the United States and the United Kingdom (UK), among other countries, is to provide evidence-based medicine to ensure high quality support (2). (2) defined evidence-based medicine as:

“*… the conscientious, explicit, and judicious use of current best evidence in making decisions about the care of individual patients. The practice of evidence-based medicine means integrating individual clinical expertise with the best available external clinical evidence from systematic research.”*

As this definition implies, decisions on diagnosing and treating diseases involves balancing the information and wisdom between what is learned from prior scientific studies and the clinical training, experience, and expertise of clinicians. Increasingly, there is movement toward more patient-centered care and support (3). This includes recognizing and honoring the knowledge, preferences, and abilities of patients as an essential part of care and prioritizing the prevention of disease, or what is called *health*care (1).

This shift toward patient-centered care and challenges to assumptions on what is evidence-based are being further influenced by digital technologies. In particular, access to digital health technologies enables individuals to gather personal health data through wearable sensors, affordable lab screenings, genetic and genomic sequencing, and real-time health tracking apps. Personal health data access creates opportunities to study health indicators 24/7, in context and in real time. These new technologies are affording new forms of information and evidence to be incorporated into the provision of care. This is especially useful for patients with hard-to-diagnose or treat diseases, for whom classic external evidence from prior clinical trials or the training and expertise of clinicians providing the support do not have sufficient information to provide an accurate diagnosis and offer actionable care. These new technologies are resulting in a growing number of informed and empowered patients (4–6). Greater access to personal health data has enabled patients to document their individual health trends and status, which contributes to their health-related decisions and interactions with their healthcare providers (4). Indeed, obtaining personal health data can provide evidentiary support in the medical diagnosing and treatment of diseases (7).

From this context, the Project Apollo cohort emerged and was organized as a non-profit entity. The Precision Healthcare Ecosystem is a nonprofit corporation registered in California with the vision that “The Doctor of the Future is One's Self.” Its inaugural program, Project Apollo, utilizes a multi-disciplinary, collaborative, and integrative care model, the “Study of Me,” to educate, enable, and empower participants to lead a personalized health journey, guided by their own quantified, evidence-based data. Project Apollo is a patient-initiated effort with a goal helping people learn to “self-study” to better understand factors that influence their health. Project Apollo provides people with access to education and experts who can facilitate increased knowledge of how to conduct self-tracking and self-experiments. The genesis of Project Apollo began with Dr. Michael Kurisu, an osteopathic physician who actively integrates digital health data and information in pursuit of more holistic care. His idea was to form a community of patients to foster active self-tracking to learn about and be better health advocates for themselves and others. This community was inspired by one of Dr. Kurisu's more prominent patients, Dr. Larry Smarr, a well-known “Quantified Self” individual who is modeling what a patient may be in the future (8). As the community has evolved, it has also incorporated other roles, including researchers who can provide support on issues such as the ethical conduct of research or conducting rigorous N-of-1 self-study and other clinicians who can provide holistic care and support in alignment with the desires and self-study results of patients (e.g., QiGong).

The purpose of this paper is to report ethnographic research on the genesis of the Project Apollo Cohort. In particular, the Project Apollo Cohort represents a concrete, real-world, patient-initiated effort that aligns with more general aspirations of patient-centered care. The results of this qualitative inquiry shed light on the motivations, benefits, and challenges experienced by this cohort, which could be instructive for understanding efforts in participant-led research.

## Methods

Between February and May 2019, we conducted an ethnography of Project Apollo and its parent organization the Precision Healthcare Ecosystem, a 501c3 umbrella organization formed by the patients to advance the goals of Project Apollo. Qualitative data were collected through a 1-h semi-structured interview and a short survey with Apollo members to capture individual motivations, challenges, and goals. These data were augmented with meeting minutes and documents describing the formation and evolution of Project Apollo. Participants gave their informed consent prior to being interviewed and the study was verified as exempt from the Common Rule by the UC San Diego Institutional Review Board. Throughout the data collection period, the research team attended multiple Project Apollo meetings and participated in conference calls with the group.

### Data Collection and Management

An inductive ethnographic approach was used to review documents that included recorded presentations, meeting minutes and organizational mission/vision statements. Semi-structured interviews were conducted with eight Project Apollo patients and the group's founding clinician. The interview questions were developed to better understand motivations and expectations as well as potential barriers and facilitators to self-study. Interviews included open-ended questions, for example:

“What role do you feel Project Apollo will play in your healthcare journey?”

“Describe for me, in your own words, N-of-1 (self-study) research.”

“What guiding principles should be upheld in participant-led research?” and,

“What steps will you take to ensure the validity of your research results?”. Interviews were approximately 60 min in duration and were digitally recorded, professionally transcribed, and inductively analyzed to identify themes and patterns as they emerged. All transcriptions were de-identified to protect confidentiality and stored in a password-protected file accessible to the research team members involved with data collection and analysis. The transcribed interviews, participant-observation field notes, and Project Apollo records were uploaded into a qualitative data analysis software program (9).

From July to August 2019, we asked interview participants to respond to a four-question survey and we received responses from six (*n* = 6) individuals. The survey was designed to contextualize the process of self-study research Project Apollo members are conducting. The survey included open-ended questions so as to not limit participant responses, including:

“What data are you collecting (e.g., sleep, pain, function, etc.)?”

“How do you collect your data (e.g., Oura ring, daily blood pressure device, self-assessment, etc.)?” “How do you record your data (e.g., spreadsheet like Excel, journal, app, etc.)?” and,

“Any additional information about your use of digital tools to support your self-study project?”.

The survey responses supported the analysis of how Project Apollo members choose to conduct self-study research and preferred methods of tracking and storing their research data.

### Analysis

All transcripts were de-identified and each participant was assigned an identification number. Interview data, including analytic memos and meeting and field notes, were imported into Dedoose and inductively analyzed. Data analysis involved an iterative process of reviewing all transcripts and supporting data by two of us (BW and CN) and then applying inductive coding to extrapolate the predominant themes (10). Initial codes were developed independently after reviewing two transcripts and then discussed to identify final codes. All transcripts were then coded by BW and further organized by major themes. To further contextualize the data, a brief anonymous survey was sent to participants to gauge experience and usage of digital technologies and mobile health apps. The results of the survey responses were analyzed and reported as descriptive statistics.

## Results

### Participant Demographics

A total of nine (*N* = 9) individuals participated in an interview, with six responding to a follow-up survey. Participants were all adults over 18 years old and consisted of six females and three males. The estimated age range was 25–75 years of age with all reporting having complete college with the majority having a graduate degree. Participants included eight (*N* = 8) patients and one (*N* = 1) clinician associated with Project Apollo.

### Major Themes

Responses were organized under the five key themes identified during data analysis: healthcare system frustration; community support; self-study/N-of-1; need for access to experts; moving from *sick*care toward a *health*care system.

What has led patients to Project Apollo is their shared frustration with the healthcare system. They receive community support that, along with advances in technology and access to health and research experts, fosters their motivation to study their health conditions through observational self-tracking and N-of-1 studies. A common theme of community support is in empowering their decisions to go forward with self-studies to supplement their healthcare decision-making. In addition to the peer-to-peer community support, they expressed the need for access to health experts and researchers in the process of their self-study research. Ultimately, their shared hope is that through the self-study research combined with advances in technologies, they will facilitate the transformation of the broken *sick*care system to a patient-centered, precision health ecosystem. Each theme is presented below and augmented with quotes from participant interviews.

#### Healthcare System Frustration

This theme is characterized by experiences with hard-to-diagnose diseases, which is a key attraction of this Project Apollo Cohort and why Project Apollo was formed. One of the most common points of contention among Apollo patients was their shared frustrations with the U.S. healthcare system.

Frustrations with the current healthcare system included how difficult it is to navigate, receiving unsatisfactory diagnoses, undiagnosed health issues, piecemeal care, high costs (“these financial burdens, they're not fair to patients” P05), and being brushed aside. A common frustration expressed was the difficult path many faced in obtaining diagnoses of their various health conditions, illustrated by this participant:

“*It's painful. It's frustrating. That journey was so difficult for me, and I am a very strong person, but those were dark, dark times…because I was in pain, things were happening to my body, and no one could tell me what was wrong, or how to fix it” (P01)*.

“*So, it's been a long journey, and it doesn't look like it's getting…like there appear to be no solutions. It's incredibly frustrating*.

*You know, people look at me and think I'm fine,” (P17)*.

Relatedly, several participants felt they were not listened to and their symptoms glossed over by health providers. For instance,

“*It's sort of been a frustration for me for a lot of years to be kind of not in sync with my providers, where I actually have – this sounds ridiculous – to tell them which tests I need them to run. They'll question that and say, ‘Well, how can you justify this, blah, blah?' I read a lot. I read studies and I read methodology and it's frustrating for me to run up against” (P02)*, and

“*If you don't know and the scientists don't know, or the scientists say, ‘Well, it's all in your head,' which is one of the things that enraged so many of us” (P04)*.

Regarding feeling brushed aside by health providers, another participant (P05) simply stated,

“*I didn't want to be doubted as a person or a patient.”*

P01 continued to describe how the common thread of frustrating healthcare inspired the formation of Project Apollo,

“*But basically, the founding cohort of Project Apollo came together because we have managed to navigate the healthcare system to begin to get the kind of care we needed, and we unanimously felt that it shouldn't be this hard.”*

“*P02: At least speaking for myself, and I know with some of the others, we find it difficult that the various specialists that we're seeing don't seem to be communicating together and/or don't seem to be, how can I say, not yet quite comfortable with the concept of collaborative care, where [it's] the patient them self who really has the most in depth understanding of how their body works.”*

Another participant analogized the broken healthcare system to that of a storybook character:

“*I was thinking about medicine as Humpty Dumpty, in that the current healthcare system has broken the patient into “parts care” via specialists, and that only through an integrated patient-centered whole person approach can we put Humpty Dumpty back together again to help patients heal and become whole again. All the king's horses and men cannot do it…we must involve the patient.” (P05)*.

#### Community Support

This theme of Community Support is perhaps the strongest predictor of how successful Apollo may be in the future. Resonating across all Apollo participants is the close community support system they have created. For patients who have experienced serious hardships in their healthcare journey, Project Apollo was often described by patients as place of solace and support.

While many participants expressed dissatisfaction with the healthcare they received, Project Apollo was explained to be a group that provides a place of support and guidance where patients can express their health desires and seek answers to the questions they possess.

“*I just don't know what the answers are for me, and I need some community to help me figure that out” P17*.

Explaining what draws the group together, one participant expressed that Project Apollo is:

“a community that brings a lot of support to one another in navigating this often-broken healthcare system, as well as deep diving into our own health and I guess promoting wellness,” (P06).

The community support and group dynamic were also discussed as providing a healthy impact on participants' wellness journey, as illustrated by P15,

“*Just being a part of this group has really helped me on my health journey,”* and

P07, “*we had this kind of group meditation and a check-in and the patients got to know each-other and I started noticing that aspects of their health got better just from that intervention.”*

A strong social support system has been demonstrated to improve health outcomes (11) as well as provide meaning in life. Participant P05 spoke of how a community bond is a crucial aspect of overall health:

“*I think the community is really important. And I think just empowering. I mean we all want to live a rich, fulfilled life and it doesn't have to be with a perfect body and perfect mind but a rich, fulfilled life. So, I think that's been a huge part.”*

Another participant declared that the key to a successful self-study lies in the community aspect,

“*There's a lot of things I want to study and how would I like to study them? Well, no way better than a community of caring people who have their own self-study, with all these amazing researchers we have accessible through this project” (P01)*.

In a stark difference to other participants who highly regard the community Project Apollo brings together. One participant (P04) expressed this as being the weakest aspect of Project Apollo and needs to be strengthened.

“*That community aspect is where we're weakest. Where there's a tight group of the original founding cohort, and then there's…if this is going to grow, the community has to be attended to. I'd say if anything, that's probably the place that needs the most work, in my mind” (P04)*.

#### Self-Study/N-of-1

**Figure 1:**
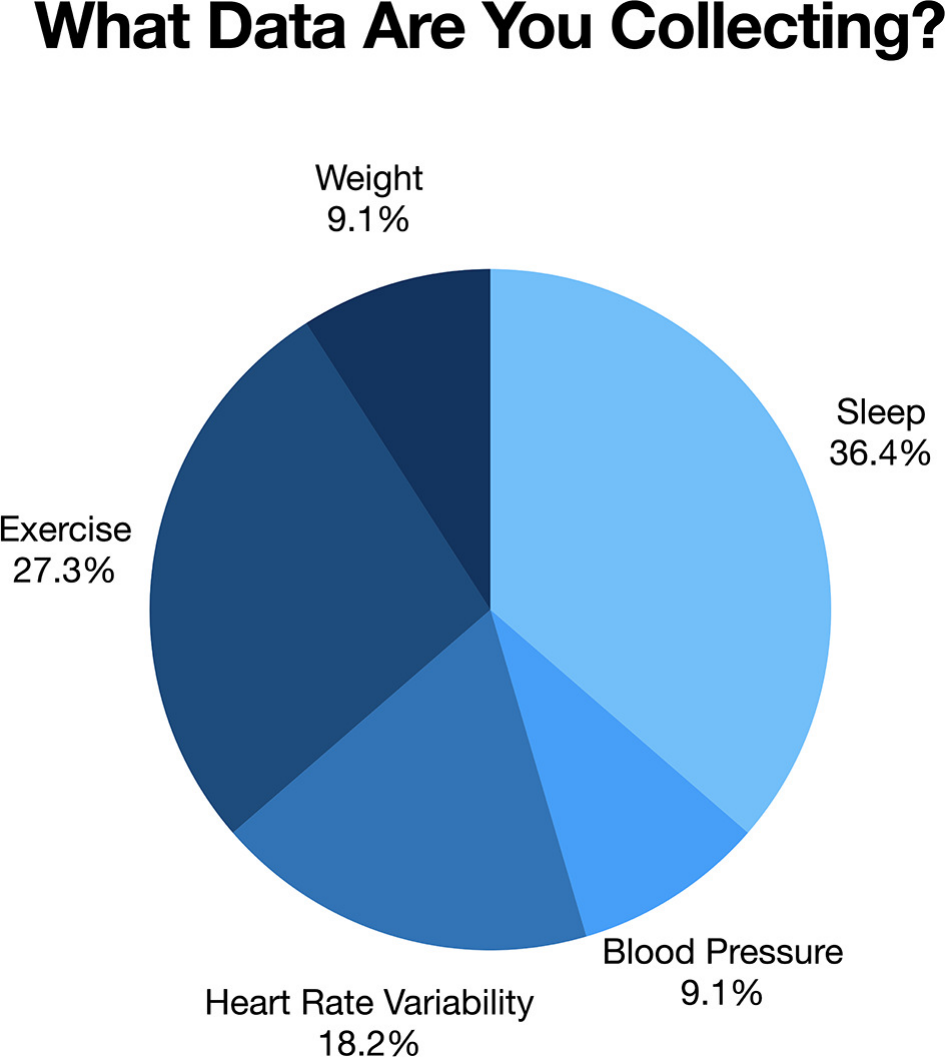
Shows health domains that Apollo participants were tracking at the time of this study.

The process of learning to self-track and carry out self-experiments plays a vital role in supplementing Apollo patients' healthcare experience. Several Apollo patients felt that without the tools currently available to assist them in conducting self-study research, they would not have been able to get this far in their health journey. In fact, our brief survey revealed that nearly all participants were using digital tools to facilitate their self-tracking process and progress. Of the nine participants who completed an interview, six individuals responded to our 4-item web-based survey. Respondents acknowledged tracking a diverse array of data, including symptoms, biomarkers, and/or physical attributes, using digital technologies including wearable sensors, mobile health apps, and real-time tracking. For example, several (five of the six respondents) had purchased an Oura ring^1^ to track information on sleep quality and activity levels. All had begun to use applications and digital health technologies to assist in tracking different health variables to inform actionable health choices (Figure 1).

A core element of the Project Apollo Cohort is the opportunity to create and implement N-of-1, self-studies. Many attributed self-tracking and self-study to their ability to take control of their health journey in a substantive way.

“*The folks at Project Apollo, I think many or most are very actively involved in their own healthcare. They've been doing a lot of their own tracking and a lot of their own finding providers that are most helpful” (P02)*.

Similarly, participant P05 stated,

“*I think giving any individual the tools to gather data in a meaningful manner that can help them make – well, the side benefit is it will help them make decisions about their own health.”*

Self-tracking and N-of-1 research has flourished and continues to expand due to the ubiquity of wearable sensors, real-time tracking technologies, and affordable lab screenings. For example, P01 stated:

“*I want the data, I don't just want to wear the watch and see the app, I want the data. Cause I want to link that data to my day, and to the stress, to the food, and to the exercise, to see what's going on and to see if I can learn something about why my blood pressure has been up for the last couple of years.”*

Participant P15 explained how technology can help track data for self-studies,

“*I'm trying to start in the basics and I really like the Oura Ring because it just does it for me. Like even when I'm thinking about, I would like to maybe do a study on radiation-induced fatigue because it just knocked me on my butt and I'm just really curious about it…Is there a way that technology can do it for me, you know?”*

Along with advances in health-based technologies, participant-led research is growing because of a *sick*care system, which historically has disregarded patient input. Armed with shared frustrations of the healthcare they received and access to tools for self-study research, patients are empowered to act on their health conditions. Describing the conjunction of these factors, P07 stated:

“*It has morphed into this idea that this group can become much more empowered rather than the medical system doing something to them, that then they have the power to act on it. I have all this data about myself, what do I do with it? And so, part of it is well what do you want to do with it? Let's create studies, let's create personalized plans for each individual.”*

Participant P01 expressed that if they were to get the health outcomes they want, they would have to take matters into their own hands,

“*So, until I got it that the only person that was going to drive my care was me and I'm not taking no for an answer, and if somebody is scratching their head, I'll find somebody that will dig deeper with me.”*

#### Need for Access to Experts

For some in the Project Apollo Cohort, this is their first exposure to learning and applying the scientific method. Many were unfamiliar with the process of forming a hypothesis and research question and the steps of designing a study that could provide meaningful data. Moreover, the process of collecting data and skills necessary to analyze data and draw conclusions from that process is not trivial. As such, many emphasized the need to be walked through the scientific method by research experts to develop the foundational knowledge needed to do self-tracking and/or self-experimentation safely and ethically.

Apollo participants also felt it was vital when in the process of learning and doing participant-led research to receive feedback from experts including researchers and clinicians. For example, as P01 stated:

“*Oh, God. I want to be handheld and walked through it every inch of the way…there's a lot of things in the digital universe that people do better than me. And I just, I know the limitations of my experience and my capabilities. And I can do things, but I just need step-by-step instructions.”*

Project Apollo has added several researchers and clinical experts to support the self-study pursuits of its members, which participants agreed is key for successful N-of-1 research. Areas participants expressed as important for access to experts included the protocol design *(“It requires…input from other valuable support people, researchers, about what you could potentially encounter” P06*), data collection *(“What are some good objective measures and what are some ways to track them?” P01*), and data analysis *(“I could look at some patterns, but I immediately need that feedback.” P05*) in their N-of-1 research studies.

To provide consistent foundational instruction about the scientific methods and responsible research practices, educational modules were adapted from the Building Research Integrity and Capacity (BRIC) curriculum developed by Dr. Camille Nebeker and made available to the Project Apollo Cohort (12). The adapted BRIC educational modules were made available online for the Cohort members to review in advance of planned face-to-face training sessions, which were designed to apply the concepts introduced via BRIC. Two face-to-face training sessions were convened to discuss the modules and begin the process of developing a research question, identifying measurement strategies, and creating a data collection and recording plan. Specific to the BRIC modules and group discussions, P04 exclaimed,

“*Boy we need the training. I know that's where, after reading the threads in Slack, I know that's the push now, the BRIC [training modules], the realization that we've got to have training.”*

Understanding of the scientific method takes time and applying the method to self-study takes practice and trial-and-error. Moreover, access to experts throughout the process was deemed critical.

“*I'm not a statistician. I think it would be great to have people we could talk to with different expertise like that who could address, especially interaction between different factors. That, I'm not at all comfortable that I know how to do that” (P02)*.

And, “*at some point that question might be, ‘how do I begin to answer this question?' and the answer to that might be, ‘seek the insight of someone skilled in x, y, or z”* (P03).

The importance of access to researchers and clinicians includes how and when to share self-study results that may indicate the need to obtain medical attention. Participant P15 stated,

“*But it's not like it's giving me information that's going to lead me to self-diagnosing and self-treating. Because I think that can be very dangerous, even with me as a nurse, like as Master's in Nursing, I don't feel comfortable doing anything without a doctor telling me to do it, especially with the cancer.”*

#### Moving From *Sick*care Toward a *Health*care System

The transformational shift from *sick*care to healthcare involves integrative and personalized medicine supported by the patient's role in self-tracking and self-study. Both clinicians and patients must be actively involved to realize this paradigm shift.

A vision and mission of Project Apollo and its parent organization, the Precision Healthcare Ecosystem^2^, is to create “a world of people empowered to realize optimal health” where the “doctor of the future is one's self” and subsequently, “transform healthcare through data-driven, patient-centered collaborative communities.” The motivation to revolutionize the healthcare system such that it is tailored to the health experience of individuals through precision medicine and patient-led self-study. As stated by P07

“*patient-led research can start driving us into a greater understanding by getting closer and closer to the unique lived and mysterious experience of each individual life.”*

To realize this ambitious goal, medical education will need to change. As stated by P02,

“*The medical education system hasn't yet changed sufficiently. I think it's changing with the existing model shifting to an individualized care model; but patients aren't in the middle of that equation; patients aren't even in the conversation. We're in a really exciting time given the technological advances, and although clinicians are experiencing a lot of burnout due to the current healthcare system - patients are experiencing patient burnout. Project Apollo provides a really great opportunity to move things forward and do what we all came here to do, which is promote health and live our best lives.”*

From a clinician's perspective, the idea of individualized care may seem intuitive, as noted by P07

“*It's not like I'm going use the same hands-on technique for every single person because their anatomy, their physiology, their life, everything is different. So, it has to be adapted. N-of-1 is the study of just one individual, and there's a lot of research right now being done on N-of-1 precision medicine and a lot of that is in the pharmaceutical grade, especially with designer drugs for cancer.”*

Clearly, our current *sick*care system is not designed to support this level of individualized care and, as such, it will continue to take a toll on both patients and clinicians as the process of transformation takes place.

The impetus of Project Apollo was to explore whether patients who were already collecting data independently could be a collective force in shifting the health ecosystem. The idea of self-tracking and self-study maybe essential to transforming healthcare; however, while the concept may seem simple, in reality it is quite challenging. Independently, the patients who became the Project Apollo Cohort were navigating the complicated waters of the current *sick*care system and had developed expertise that collectively could help others avoid the frustrations they had experienced. Some of this expertise was in knowing what questions to ask and of whom, but it was also synthesizing the corpus of medical information amassed from various tests across a multitude of clinicians. Self-tracking, while it may not have been systematic or even labeled as such, was inherent to the Apollo Cohort members. As P02 recalled,

“*this whole idea is a foundational part of our goal to generalize to large communities and perhaps people who know nothing about this or weren't aware of or have never maybe done a deep dive, reflective, introspective analysis of their own health and what could be better and things like that.”*

The process of learning a more systematic approach to self-tracking involved learning new methods of collecting and making sense of personal health data, including new vocabulary. In P04's case, the phrase “*precision medicine*” was not familiar, and there was an excitement about taking control as expressed in this quote:

“*Project Apollo, for us, represented an intriguing intellectually interesting endeavor…and a chance to break out of the limitations, get away from these predictions, get away from the statistics, and get into a level of medicine that's really about you, and not confined to a rushed 20-minute appointment.”*

## Discussion

Digital health technologies and mobile health applications are integral to the success of Project Apollo self-studies and empowering patients in their health making decisions. In addition, peer-to-peer support and the creation and sense of being part of a community are also essential aspects of this work.

### Implications for Patient-Centered Care

These results point to the possibility of patients not merely being “empowered” by professionals, but also taking leadership roles within their own care and, alongside professionals, advancing peer-to-peer support to one another. This has important implications both for understanding the role of patients in the health sciences and also on the future of care, particularly the active integration of peer-to-peer support.

With regard to the role of patients within health sciences, the Project Apollo Cohort could be viewed as a form of citizen science. Citizen science is an umbrella term with origins in the disciplines of ecology, ornithology, and astronomy that have involved the public in conservation and crowdsourcing (13). More recently, citizen science has moved into the health sector (14). As in other fields, within the realm of health, citizen science encompasses a very broad array of activities (15, 16). On one end of the spectrum, citizen scientists are involved in providing support to research efforts via volunteering their time and interest toward a well-specified and prescribed task established by researchers (17). For example, researchers have developed Fold-It^3^, a “game” that enables people to work through the “puzzle” of finding different ways that amino acids/proteins can fold over on themselves to create different types of protein structures; it is a topic that is vital for understanding issues such as antibodies and care (18). On the opposite extreme are citizen-/patient-led efforts whereby the priorities, work, and efforts are completely driven by and for the persons experiencing the issue. For example, the #WeAreNotWaiting^4^ community of patients with type I diabetes is a self-organized, highly networked, modular group of individuals with type I diabetes (or parents of children with type I diabetes) who found ways to drive advancements in their self-care (19). Some concrete examples of solutions that grew out of this community include Nightscout^5^, an open source tool used to gain access to a patient's continuous glucose monitor data, and the Open Artificial Pancreas System (OpenAPS)^6^, which, building on Nightscout, is a closed loop artificial pancreas system algorithm created via self-motivated patients and those who care for them (20). In between these two extremes are truly collaborative efforts in which power and agency is shared between traditional professionals and patient/citizen scientists. For example, the Robert Wood Johnson Foundation-funded Opening Pathways Project^7^ was a research effort led by Principal Investigator (PI) Dana Lewis, a patient innovator who leads the OpenAPS community, with traditional professors playing roles of Co-PIs, Hekler & Johnston. The focus of that project was on advancing new pathways for non-traditional researchers to advance the care and health of patients. Across all of these domains, it is common for a community of individuals with shared interests, passions, or needs to come together to work toward a shared future vision of health.

Based on the wide range of ways in which citizen science manifests, from citizens supporting researchers to citizens running efforts without any traditional professional support, there are also a wide range of methods and tools used to advance these efforts. For example, Fold-It involves robust use of data informatics, human-centered and game design expertise, and robust knowledge on surfacing difficult and intractable challenges in understanding proteomics to be combined into a fun, engaging, challenging “puzzle” that any person interested in solving puzzles can engage in. The OpenAPS community, on the other hand, uses a mixture of techniques such as open source software development practices (e.g., robust use of GitHub), community “tuning” strategies^8^ for iteratively and rigorously identifying and vetting assumptions related to any technologies developed by the OpenAPS community to ensure they are safe^9^, and also open science practices^10^ related to data sharing, data science best practices, and open data repositories, such as those supported on the Open Humans service (21).

Turning now to Project Apollo, the Apollo cohort are engaging in a wide range of hypothesis-driven “small data” approaches (22). There are a wide range of methods that fit into a small data paradigm. On one extreme, there are methods that are simple for most people to use and engage with, such as journaling and gathering of qualitative data. The value here is that most people can do it, but it may not necessarily produce as rigorous results in terms of inferring and predicting future responses of individuals. In the middle are quantitative self-tracking, more formalized hypothesis testing within an individual's time series, and non-randomized single case experimental studies used to glean insights on the impact of different decisions. These balance ease with rigor. At the more rigorous end, when very specific and concrete questions are being asked, are randomized N-of-1 cross-over designs meant to test the influence of various actions (e.g., taking a medication or not, choosing to eat a certain food or not) on targeted outcomes to use of system identification, which is a technique used by control systems engineers to identify computational models of complex, dynamic phenomena. The Apollo cohort appears to be engaging in almost all except the most extreme in terms of technical requirements (system ID) to advance understanding, as well as the quality of their own health.

The Project Apollo cohort, coupled with broader trends toward patient-centered health, points to the potential value and need for further advancing a small data paradigm to better support patient self-study. By self-study we mean the use of these small data methods by a person to help them better understand themselves toward achieving self-defined goals. Within a small data paradigm, success is defined for each person, such as reduction in symptoms, improved function, or increased self-understanding of one's own condition. By extension, this enables a clear alignment on the self-interests of patients/persons experiencing a condition, clinicians, and researchers. They also create space for different ways of knowing and understanding a person's health condition than is common from traditional evidence-based practice. Specifically, as the definition of evidence-based practice (provided at the beginning of the paper) suggests, it relies heavily on generalizable knowledge gleaned from the scientific literature and prior individuals coupled with the clinical expertise of clinicians to translate that wealth of knowledge into personalized recommendations and steps forward for each patient. This classic approach provides little structure or place for incorporating knowledge and insights from the person themselves experiencing a condition and their self-studies. A small data approach provides a structure for honoring the unique knowledge and insights self-study can bring into advancing decision-making around health issues.

As is likely obvious when looking at this spectrum of small data methods, the amount of training and prior knowledge needed to use the methods is one key tradeoff (e.g., journaling can be done by practically anyone; system ID requires deep specialized knowledge in mathematics, programming, and understanding of robust study designs to systematically test computational models). The complementary tradeoff, of course, is the capacity for these various methods to provide more rigorous insights from data for guiding thinking and decision-making related to complex phenomena (e.g., journaling has a higher risk of drawing spurious conclusions compared to N-of-1 cross-over trials or system ID studies). Based on this, a key implication from the work of the Project Apollo cohort is the need for a wide range and diversity of training materials (e.g. tailored education) and resources (e.g., health coach, professional researchers) that support the many different ways in which patients may engage in self-study, from basic journaling to rigorous predictive mathematical models designed for each person. This is not only the case for the patients themselves, but also points to the need for professionals, particularly clinicians, to learn how to understand, honor, and integrate this type of evidence into their clinical practice and support. It also may point to a new type of healthcare service, a self-study coach, who, alongside a health coach, physician, nurses, and others on the care team, could take the time to help individuals engage in appropriate self-study to facilitate self-learning and not over-generalize results, either to themselves and definitely not to others, as doing N-of-1 study does not, alone, produce transportable knowledge (22).

Moving now to the stated importance of community that emerged from the Project Apollo cohort, this work points to the possibility that “patient-centered” may, in fact, be too limiting of a concept. In particular, the Project Apollo members clearly highlight ways in which support and care can, and perhaps should, be offered that go well beyond the traditional dyadic relationship between patients and their providers, or even patients surrounded by providers. Indeed, the work points to the value patients receive when they can work and discuss their experiences with other peers. While “care” has always been identified within healthcare, in many ways the desires, interests, and active cultivation of a caring community of patients highlights that the professionalization of care may not be adequate, or even appropriate, compared to what people need. This fits with broader trends and interests in peer-to-peer support, such as the work of Susannah Fox^11^ in supporting peer-to-peer advice online and Rajiv Mehta advancing an “Atlas of Caregiving^12^,” whereby individuals learn to understand and cultivate care and caring within and across families and communities.

As with self-study though, peer-to-peer requires further reflection and training. For example, a key risk of peer-to-peer support involves a person translating personal history and beliefs on what was helpful for them into explicit recommendations of activities that others should engage in. As health sciences, writ large, demonstrates, it is no small task to determine if a recommendation is indeed an appropriate, safe, and effective recommendation for others to use. Peer-to-peer is not an appropriate venue for offering treatment recommendations and the like, as the underlying epistemology does not support that type of offering. With that said, peer-to-peer support does offer a place for care, warmth, and shared experiences to be communicated. Furthermore, peers can feasibly be excellent sounding boards for one another to help each other think through plausible pathways forward on a given condition, particularly when determining the right diagnostic, prevention, treatment, and health promotion options are unclear. In this domain, peers can share their stories and experiences and, grounded in a shared recognition of individual choice and agency, patients can then engage in balancing their understanding with what they might learn from self-study, their clinician, or the external evidence-base. It is these latter benefits of support, shared experience, and what not that the Project Apollo group is seeking from one another, not evidence-based recommendations. Based on this, future work points to the need for further understanding, reflection, and integration on how patient peers can and should interact with one another, their care team, and broader community members (e.g., health scientists and ethicists with expertise that could be valuable for patients to think through their self-study).

**Figure 2:**
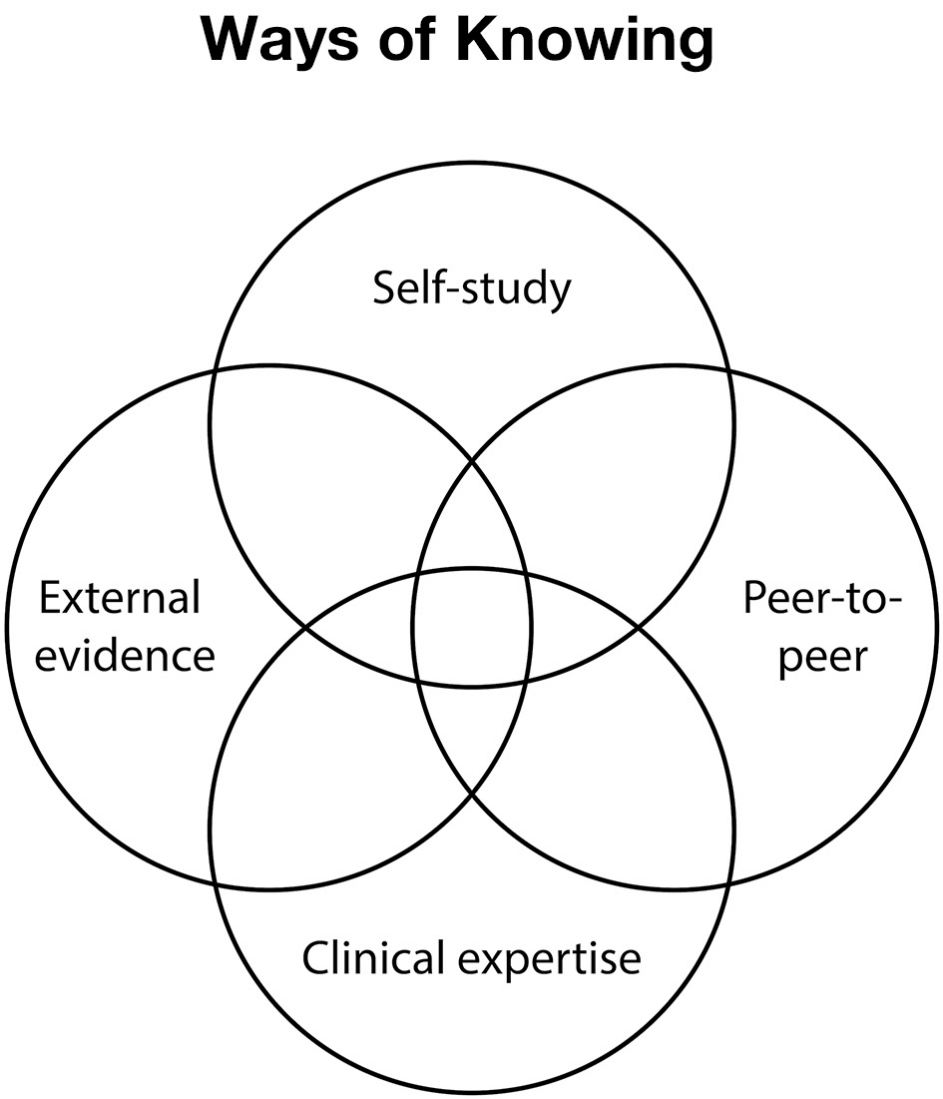
Depicts four plausible ways of knowing presented as a Venn Diagram with external evidence, clinical expertise, self-study, and peer-to-peer support forming a foundation for a new type of evidence-based medicine.

Overall, this work points to two ways of knowing and advancing ones' health that fall outside of the realm of the external evidence from the health sciences and clinical expertise. As such, the work of Apollo acknowledges potential limitations of the classic definition of evidence-based medicine. This work points to four plausible ways of knowing: external evidence, clinical expertise, self-study, and peer-to-peer support, as a possible foundation for a new type of evidence-based medicine, what might be thought of as evidence-based practice 2.0 (see Figure 2). Each of these ways of knowing have different strengths and limitations that, when combined, are highly complementary. For example, classical health science provides a robust “warm start” that enables people to quickly rule out different types of diagnoses and treatment options (22). Clinical expertise provides insights on patterns of responses across the many patients that clinicians see to further improve decision-making and rule out different diagnoses, treatments, and actions. Self-study provides a structure for enabling a person to identify and test assumptions around diagnosis and treatment specifically for themselves. Finally, peer-to-peer offers insights on plausible hypotheses, beliefs, and coping strategies that are not yet well-studied or understood in the scientific literature or part of clinical practice. When used together, these four references balance out the relative strengths and limitations of one another toward more robust, personalized decision-making.

There is a great opportunity to improve care if robust approaches to self-study (alone, in partnership between patients and clinicians, and even partnerships between patients, clinicians, and researchers) and peer-to-peer support can be defined. As alluded to, there is an opportunity to provide complementary knowledge and insights to that which is offered from traditional scientific methods and clinical expertise. There is also the opportunity for improving communication and understanding between the lived experience of persons and clinicians seeking to support them. This type of approach could also provide a foundation that enables patients to feel more capable of understanding themselves and finding solutions for their personal health needs. Finally, if these four references could be established as working synergistically together, they could enable new insights, ways of thinking about health and care, and strategies for improving health to emerge.

While the opportunities are great, there are also a myriad of challenges that this vision of care implies. At the most basic level, this type of approach, to the best of our knowledge, does not yet exist. Specifically, we are unaware of any health organization that actively and consciously balances knowledge, skills, and expertise across external scientific evidence, clinical expertise, self-study, and peer-to-peer support. This means that new skills, training, and even mindsets for all relevant stakeholders (e.g., patients, providers, researchers, and payers) are likely needed. Ideally, this training would focus on ensuring appropriate conclusions are drawn from each referent. For example, external scientific evidence produces “on average” insights, but that does not necessarily equate to an individual; in contrast, self-study produces insights that could be valuable for a person, but that does not mean those insights would be transportable to anyone else. Furthermore, traditional clinical training is focused on providing a clinician with a structure for calibrating between the scientific literature and patterns they have observed over time among their patients; this means that clinical expertise and intuitions could be inappropriate to be relied upon if either there is little research on something a person is experiencing or if a clinician has little prior personal exposure to other patients experiencing a given phenomenon. Finally, peer-to-peer provides a way for individuals to explore different ways of understanding themselves in context and develop alternative beliefs around health, but those alternative perspectives, even if appropriate for a given group of people, are not necessarily appropriate for others; thus, peer-to-peer can be thought of as a great venue for generating new hypotheses and strategies to move forward, but not as a venue for testing ideas or gaining reliable, rigorously vetted recommendations. New approaches that support this calibration are needed, along with appropriate training and guidance on this work.

Beyond basic use of the methods, this approach also introduces new challenges to traditional approaches for considering ethical practices and, by extension, appropriate oversight and regulation. Traditional regulations largely rely on the implicit assumption that the professionals generating scientific evidence and clinical expertise have the requisite information needed to guide ethical decision-making. Moreover, federal regulations for the protection of human participants in research do not align with participant-led self-study forms of citizen science (4, 23). Based on this, the activities for regulating self-study and related ethical practices largely conform to the monitoring and regulation of professionals and their practices, leaving this novel form of citizen science unregulated. That is, individuals engaging in self-study and peer-to-peer support are by definition working outside the realm of existing regulation, and it is unclear the extent to which existing principles for the ethical conduct of research pertains (4). There is a clear need for strategies that enable an individual patient/person to conduct an ethical self-review, including assessment of the potential risks and benefits, to reduce the likelihood of negative unintended consequences either to oneself or to others. Furthermore, for peer-to-peer, there is a need for structures that enable checks on beliefs and also appropriate practices on what is appropriate vs. in appropriate in terms of peer-to-peer support. For example, in peer-to-peer circumstances, it is appropriate for people to share stories and current thinking on what they are doing and how they think their actions are resulting in positive changes. It is inappropriate for peers to engage in providing advice and recommendations, particularly if current beliefs are largely grounded in one's own experience. As we develop these practices, key lessons can be learned from how open source efforts, such as Wikipedia, function in terms of governance (24) as, almost by definition, an overly top-down regulatory process will not only be insufficient, it is likely inappropriate for this type of work. Interestingly, insights from philosophy of science, particularly on thinking through ways to develop trustworthy scientific consensus in ways that do not use top-down structures of regulation, could be another starting point for inspiration on different types of ethical practices (25).

## Open Questions and Limitations

We conclude our discussion with five provocative questions along with limitations to our study.

1. How can clinicians support patient agency? Clinical support for agency and autonomy for patients is fundamental in the practice of medicine but has been proven to be a very challenging and difficult task (26). The healthcare system that we are all a part of does not seem to be set up to allow this to happen naturally. Paternalism, whether good or bad, is a pervasive attribute that exists within the culture of practice of medicine. It is imperative that we look at this as we try to form a new paradigm of the doctor-patient relationship.
2. What are the barriers and facilitators to clinician support? It is essential that we consider a complete and holistic view of the patient. This includes all areas and aspects of patients' lives that impact health and wellness, including social determinants such as socioeconomic status, community, family dynamics, race, and culture. This gives a broader and more rich contextual relational understanding of the patient (27). Unfortunately, a holistic perspective is not emphasized and modeled enough in medical school, nor is the importance of the patient's role within the doctor-patient relationship included in the curriculum.
3. What is needed to develop clinician education to advance precision healthcare that involves patients throughout the process? Perhaps a more important factor regarding this topic is to take a broad holistic view of the healthcare system. The current system is multifaceted in complexity, and over time it seems to have undermined the doctor-patient relationship (28). There is over-reliance on information technology, medical devices, and procedures and less time spent nurturing empathy, compassion, and connection with the patient (29). The result is a growing distance in the doctor-patient relationship and a mirrored discontent amongst both participants (30).
4. How to foster autonomy in patients when one of the most common frustrations from the clinicians themselves is their own feeling of lack of autonomy within the healthcare system. Physician burnout is at an all-time high and its consequences are disastrous (31). On many measures, the actual clinicians have worse healthcare outcomes than the patients they are treating (32). This, in turn, creates a downward spiral negatively effecting the entire system (33). As designed, the healthcare system is not sustainable. It is time for change as the system needs to be designed to care for all involved.
5. What is the vision of Apollo moving forward? The essence of Project Apollo is a co-creative and collaborative nature of building community amongst patients, providers, and researchers. The cohort also prototyped different ways of experimenting with enhancing patient autonomy and agency. This will be accomplished through educational modules, self-tracking, retrospective analysis of healthy behavior changes, and utilizing technology not as a barrier, but as a tool to augment the connection the patient has with their physician. This can create a new paradigm empowering patients and physicians to have a more enhanced doctor-patient relationship. A co-creation of a new model of the way care is delivered can be designed benefiting all those within the healthcare system.

There are limitations to this study. The Apollo Cohort consists of a small group of about a dozen patients and, while those who agreed to be interviewed (*N* = 9) represent a majority of the group, it is not appropriate to view these data as representative of self-study as it relates to patient-centered care. In qualitative research, a goal is to reach saturation of the data to have confidence about the phenomena under study and, while our sample was a representation of the Apollo Cohort, the themes identified may not be generalizable to others involved with self-tracking or participant-led N-of-1 studies. Another consideration is that we, the authors, have been involved in discourse with the Project Apollo Cohort from the early days of its formation and it is feasible that we have influenced the community and their conceptualization, just as they have influenced us. To mitigate the potential bias introduced, we followed best practices in qualitative research, such as including several data sources to inform our finding, involving two researchers coding the data, and engaging participants and peers in the review of our results. Given the novelty of patient/participant-led research and how it presents (e.g., DIY, lead innovators, Quantified Self), we believe the potential value in learning from the Project Apollo cohort experiences is noteworthy with respect to self-study and potential impact on the current sick/healthcare system.

## Conclusion

Our study revealed how N-of-1 research, in the form of self-tracking and self-study plus communal support, can contribute to one's health journey and create a pathway for active collaboration in advancing precision healthcare. Facilitators to engaging patients in self-study include motivation, albeit stemming from frustration, and a safe community where support is derived from one another. Additional support in the form of access to experts who can help with important foundational knowledge necessary to conduct meaningful self-study is critical. Moreover, increasing awareness of healthcare professionals about the potential value of collaborating with patients who are advancing health knowledge through self-study will be a key factor in the success of patient-centered care and in shifting the paradigm from *sick*care to collaborative *health*care.

## Data Availability Statement

The datasets used for this study are qualitative and will be available via the Qualitative Data Repository (https://qdr.syr.edu/) once redacted to protect the identity of participants.

## Ethics Statement

This study involved human participants and was reviewed and verified as exempt by the University of California San Diego, Institutional Review Board. Participants provided their verbal informed consent prior to being interviewed.

## Author Contributions

CN conceptualized the study and contributed to data analysis, writing, and editing of the manuscript. EH contributed to writing and editing the manuscript. BW contributed to data collection, analysis, and writing and editing the manuscript. MK contributed to writing and editing the manuscript.

## Conflict of Interest

The authors declare that the research was conducted in the absence of any commercial or financial relationships that could be construed as a potential conflict of interest.

## Footnotes

^1^ https://ouraring.com/

^2^ https://precisionhealthcareecosystem.org/

^3^ https://fold.it/portal/

^4^ https://www.healthline.com/health/diabetesmine/innovation/we-are-not-waiting#1

^5^ http://www.nightscout.info/

^6^ https://openaps.org/

^7^ http://openingpathways.org/

^8^ http://openingpathways.org/communal-tuning

^9^ http://openingpathways.org/is-it-safe

^10^ https://opensource.guide/best-practices/

^11^ https://susannahfox.com/

^12^ www.atlasofcaregiving.org
